# A Community-Based Cross-Sectional Study about the Knowledge, Attitude, and Practices of Food Safety Measures among Rural Households in Bangladesh

**DOI:** 10.1155/2022/7814370

**Published:** 2022-09-30

**Authors:** Mst. Rokshana Rabeya, Md. Hasan Bin Zihad, Md. Anis Fakir, Most. Sabina Khatun, Jinnat Jahan Rakhi, Ashraful Islam, Rashedul Islam, Md. Abdullah Saeed Khan, Mohammad Delwer Hossain Hawlader

**Affiliations:** ^1^Department of Public Health Nutrition, Primeasia University, Dhaka, Bangladesh; ^2^National Institute of Preventive and Social Medicine, Dhaka, Bangladesh; ^3^Department of Public Health, North South University, Dhaka, Bangladesh

## Abstract

**Background:**

Food handlers have been found to play essential roles in transmitting foodborne diseases and can pose a significant public health problem. Our study aimed to assess the knowledge, attitude, and practices (KAP) of food safety measures among the rural households of Bangladesh.

**Materials and Methods:**

We conducted this community-based cross-sectional study among women above 18 years involved with food preparation in rural households of four villages in Bangladesh. A total of 400 respondents were selected using the multistage cluster sampling technique. Data were collected using pretested and predesigned questionnaires based on the World Health Organization's (WHO) five keys for food safety. We used Stata (Version 16) for all statistical analyses.

**Results:**

The mean age of the participants was 42.09 ± 12.96 years. The median KAP scores [interquartile range (IQR)] were 7 (21–10), 16 (5–18), and 26 (9–30), respectively. We found the median KAP scores were significantly lower in the age group >55 years than in age groups of 18–25, 26–35, 36–45, and 46–55 years (*P* < 0.05 for all). In addition, the median KAP scores were significantly higher in respondents who were married, literate, employed/active, living in pakka/semipakka house, and with a monthly family income of >5,000 BDT (*P* < 0.05 for all). Among all, 33.75%, 80.25%, and 69.00% had good (≥80% of total) KAP scores, respectively. Multivariable regression analysis revealed that monthly family income >5,000 BDT was a significant predictor of good knowledge [Adjusted Odds Ratio (aOR): 3.51, 95%CI: 1.55–7.98], good attitude (aOR: 5.82, 95%CI: 2.80–11.70), and good practice (aOR: 3.18, 95%CI: 1.67–6.07). Age >55 years was a significant predictor of good attitude (aOR: 0.38, 95%CI: 0.17–0.81) and good practice (aOR: 0.48, 95%CI: 0.21–0.89). Having ≤4 members in the family was a significant predictor of good practice (aOR: 1.85, 95%CI: 1.13–3.03) regarding food safety measures.

**Conclusion:**

The study found that KAP among rural Bangladeshi women regarding food safety were relatively satisfactory. However, having a poor monthly income and living in a large family were impediments to good food-safety practices where work can be done. The findings of this study may help develop health intervention programs for food handlers to further improve KAP toward food safety, thereby reducing foodborne illness in households.

## 1. Introduction

Food is a vital necessity for the growth and development of the human body. However, it might get contaminated in touch with water, air, dust, equipment, sewage, insects, and rats and due to inadequate hygiene of food handlers. Food remains one of the major sources of infectious agents for the human body due to improper practices in its production, preparation, and handling [1]. Foodborne illness might result in an outbreak if two or more cases of infection ensue after eating a common food [2].

Food handlers play a major role in ensuring food safety during the process of food manufacture and storage [3]. They are often responsible for spreading pathogens passively from polluted sources such as uncooked meat to ready-to-eat meals. They might harbor and disseminate foodborne pathogens such as hepatitis A, norovirus, *Salmonella typhi*, *Staphylococcus aureus*, and *Shigella* spp. in their hands, face, skin, and skin hair, especially if there are cuts or wounds [4]. *Escherichia coli* O157 : H7 and nontyphoid *Salmonella* may spread from food handlers while recovering from infections [5]. Food hygiene includes all conditions and steps required to ensure the safety and suitability of food at all stages of food production [6]. Good food hygiene can help prevent food contamination and, thereby, infections.

It is estimated that more than one-third of the world's population suffered foodborne illness [7, 8]. If food handlers take care to clean their hands, bodies, and clothing during cooking, it will prevent cross-contamination of produce. Hence, knowledge, attitude, and practices (KAP) of food hygiene among handlers are essential factors in preventing spoilage of staples by microorganisms. Studies have emphasized the need for education and training on food hygiene among those who handle food during any part of preparation [9–11]. As foodborne diseases are on the rise in both developed and developing countries [12, 13], food safety measures are essential.

Personal hygiene is critical in preventing contamination of food and foodborne illness, and proper application of the “Five keys for Food Safety” from the World Health Organization (WHO) [14] helps combat foodborne illness. A study among institutional food handlers of Ghana indicated that more than three-fourths understood the importance of general hygienic measures such as hand washing (98.7% accurate answers) and using gloves (77.9%). In contrast, knowledge about foodborne disease transmission was poor in that 76.2% and 70.6%, respectively, of the food handlers did not realize that *Salmonella* and *Hepatitis A* were foodborne pathogens [15]. According to evidence from Kenya [16], Portugal [17], and Saudi Arabia [18], knowledge of food safety and proper food handling techniques resulted in improved food safety measures.

Previous studies conducted in Bangladesh regarding food safety knowledge and practice among individuals mostly involved food handlers in restaurants, meat vendors, chicken vendors, and baking factory workers [19]. Their knowledge and practice regarding food safety were found unsatisfactory. The only study conducted among household food handlers in two major cities of Bangladesh reported good knowledge and practice of food safety [20]. However, in rural Bangladesh, women are responsible for cooking in most houses. Hence, the present study was undertaken to assess KAP about food safety among women of rural households based on the WHO's five keys for food safety.

## 2. Methods

### 2.1. Study Design, Settings, and Population

We conducted this community-based cross-sectional study between January 2021 and June 2021 in four villages, namely, Maloncho, Bokjuri, Andarmanik, and Chor Bewtha, in the Manikganj district of Bangladesh. Women aged above 18 years who were mainly involved in food preparation in the households regularly were approached for inclusion. Those who declined to give consent were excluded.

### 2.2. Sample Size, Sampling Technique, and Participants

**Figure 1:**
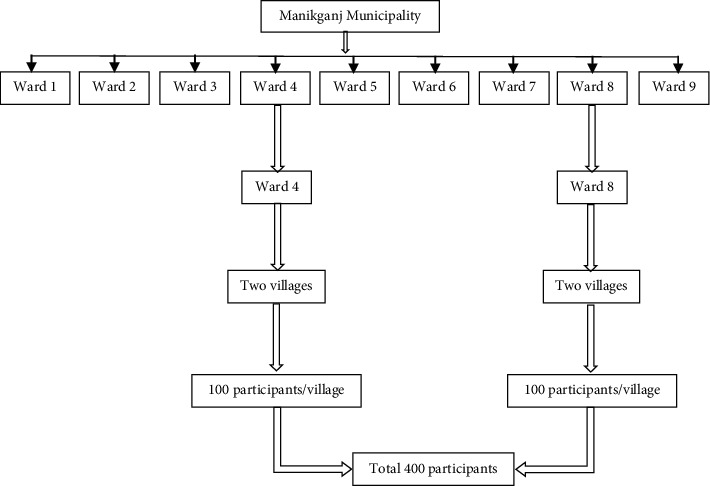
Multistage cluster sampling.

Considering 50% population prevalence (as the original population prevalence or any estimated prevalence of knowledge, attitude, or practices relating to food hygiene was unknown), 5% error, and 95% confidence interval, our sample size was 384. We included 5% extra samples to compensate for potential nonresponse. Hence, our final sample size was 403, but we kept the size to 400 for the study. We collected data through a multistage cluster sampling technique (Figure 1). First, nine wards from the Manikganj district were taken as a cluster. Then, two wards were randomly chosen from them. In the second stage, four villages were randomly selected from those wards (two villages from each ward). Finally, we selected households from each village using systematic random sampling and took every fourth household. From each household, all women involved in daily food preparation were approached and were considered food handlers. In case of nonresponse, we approached the next fourth household from the village. A total of 100 samples were taken from each village.

### 2.3. Data Collection Tools

A semistructured questionnaire was used for data collection. The first part of the questionnaire obtained information on the sociodemographic characteristics of the participants. The second part contained the WHO questionnaire [14] to assess KAP regarding food safety. In the knowledge section, there were 10 items to assess food safety. The response to each item was “True” or “False.” One mark each was given for each correct answer, and zero mark was given for each wrong answer with a maximum score of 10. There were ten items to assess the respondent's attitude toward food safety. However, one item (“Meat thermometers are useful for ensuring food is cooked thoroughly”) was removed because this is not commonly used in the country. Each item was assessed using a 3-point Likert scale: “agree,” “not sure,” and “disagree,” and the corresponding marks were two, one, and zero, respectively, totaling a maximum score of 18. Similarly, 10 items were included to assess the respondent's food safety practices. Each item was assessed using four options, “always,” “most of the time,” “occasionally,” and “never,” marking 3, 2, 1, and 0, respectively, with a maximum score of 30. After obtaining written informed consent, data were collected through face-to-face interviews. A translated Bangla version of the questionnaire was used for data collection.

### 2.4. Statistical Analysis

Descriptive statistics were considered to represent frequency, percentage, mean, and standard deviation. Data distribution was checked using a histogram with a density curve and the Shapiro–Wilk test. Comparison of KAP scores was done using the Mann–Whitney *U* test and Kruskal–Wallis test with *post hoc* analysis using Dunn's test wherever appropriate. KAP scores were categorized using Bloom's cut-off point of 80%. A score equal to or above 80% was considered “good” and that below 80% was considered “poor.” Univariate and multivariable logistic regression analysis was conducted to explore factors associated with good KAP scores. Pairwise correlations among these measures were carried out using Spearman's rho (*ρ*) test. A *P*-value <0.05 was considered significant for all tests except for Dunn's test, where a *P*-value <0.025 was considered significant. Data were analyzed using the Stata (Version 16). Graph was created using R Studio (Version 2022.07.1). Mendeley (Version 1.19.8) was used to create the style of references required for submission to the journal. The attitude and practice questionnaire, which used a Likert response scale, showed good reliability among our participants (Cronbach's *α* = 0.823 and 0.844, respectively).

### 2.5. Ethical Consideration

This study was approved by the Ethical Review Committee of the Department of Public Health Nutrition, Primeasia University, Dhaka, Bangladesh (Memo no. PAU/IEAC/22/111). Written permission was taken from the Mayor of Manikganj Municipality. The procedures were conducted in accordance with the Declaration of Helsinki. In addition, informed written consent was obtained from each participant before inclusion.

## 3. Results

The average age of the 400 participants included in this study was 42.09 ± 12.96 years (SD). Of all, the majority (28%) of the respondents were in the age group of 26–35 years, followed by 24.3% in the age group of 36–45 years. Most participants were housewives (54.5%) and married (80.3%). Of all, 35.5% were illiterate and 44.5% had a primary level. The majority (83%) of the participants were from nuclear families and had four members in their family (35.5%). Respondents most frequently had “Kacha” houses (72.3%) but “Pakka” toilets (96%). The socioeconomic status of the study subjects based on their monthly income showed that 46.8% of respondents had family earnings above 10,000 BDT, 38.3% had 6,000–10,000 BDT, and 15% had below 5,000 BDT (Table 1).

**Figure 2:**
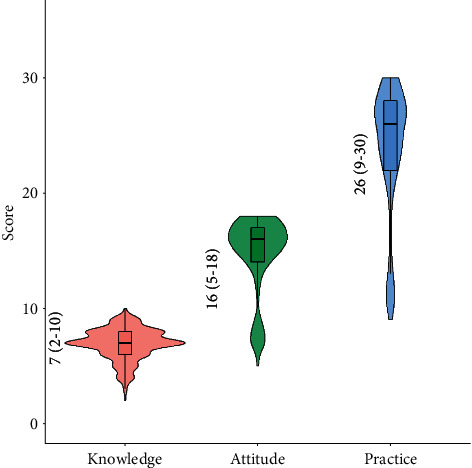
Violin plots showing the distribution of knowledge, attitude, and practice scores among participants. (The numbers shown on the left side represent the median (IQR). Note that the possible score range of knowledge, attitude, and practices are 0–10, 0–18, and 0–30, respectively.)

The distribution of the KAP scores among the study participants is shown in Figure 2. The median [interquartile range (IQR)] KAP scores were, respectively, 7 [2–10], 16 [5–18], and 26 [9–30], indicating most participants had an average knowledge score but above average attitude and practice scores.

Table 2 shows the knowledge scores of the participants per WHO's five keys for food safety. Almost 99% of the participants knew that their hands must be washed before handling food, and 88.8% knew that wiping cloth can spread microorganisms. However, 64.3% of participants knew that the same cutting board cannot be used for raw and cooked food. Of all, only 12.5% identified that “cooked meals can be left at room temperature” is a false statement. However, 83.3% knew that refrigerating food slows bacterial growth. Only 26.8% of the participants knew that safe water cannot be identified by the way it looks, but almost all respondents (99.3%) knew that fruits and vegetables should be washed before eating.

Table 3 describes the attitude scores of the participants per WHO's five keys for food safety. Among the 400 participants, 77.3% agreed that frequent hand washing during food preparation is worth the extra time. However, 17.8% of participants were unsure whether keeping the kitchen surface clean could reduce the risk of illness. Among the respondents, 69.8% agreed that keeping raw and cooked food separate helps prevent illness, and about 77% agreed that soups and stews must be boiled to ensure food safety. In contrast, only 55% of participants agreed that thawing food in a cool place is safe. Most of the respondents (96%) agreed that food inspection for freshness and wholesomeness to ensure food safety is necessary. The majority (93.5%) of the participants agreed with the importance of throwing food beyond the expiry date to ensure food safety.

Table 4 depicts the practice scores of the participants per WHO's five keys for food safety. Among all, 90.8% always washed their hands before or during food preparation. Only 40.3% of participants always reheated cooked food until it was piping hot, and 14.2% of respondents never practiced storage of leftover cooked food in a cool place within two hours. Nevertheless, 92.5% of participants always washed fruits and vegetables with safe water before intake.

The median KAP scores of food safety were significantly lower among those aged >55 years compared to those aged between 18 and 25 years, 26 to 35 years, and 36 to 45 years (*P* < 0.025 for all) (Table 5). These were significantly higher in respondents who were married, literate, employed/active, living in pakka/semipakka houses, and with a monthly family income of more than or equal to 5,000 BDT compared to those who were single, illiterate, unemployed/inactive, living in Kacha houses, and with a monthly family income ≥5,000 BDT (*P* < 0.025 for all). The only exception was the practice score, which did not vary between house types (*P*=0.134). In relation to the number of family members, only the practice score varied where participants with a family member of ≤4 had a significantly higher score than those with a family member >4 (*P*=0.025). None of the KAP scores varied in relation to the family type.

**Figure 3:**
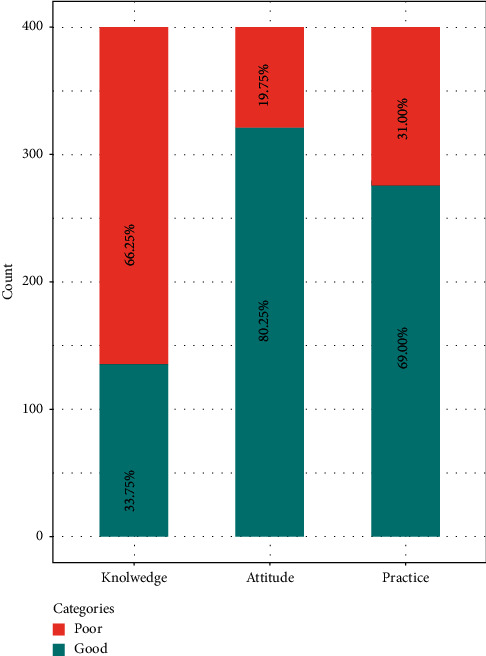
Distribution of knowledge, attitude, and practice categories among respondents.

Participants with more than or equal to 80% score in KAP were considered to have good scores. Among all participants, 33.75% had good knowledge, 80.25% had good attitude, and 69.00% had good practice regarding food safety measures as assessed by WHO's five keys for food safety questionnaire (Figure 3).

Multivariable logistic regression analysis found that a monthly family income of ≥5,000 BDT was associated with being 3.51 times (95%CI: 1.55–7.98), 5.82 times (95%CI: 2.89–11.70), and 3.18 times (95%CI: 1.67–6.07) more likely to have good knowledge, good attitude, and good practice regarding food safety, respectively. Age >55 years is associated with lower odds of having a good attitude (aOR: 0.38, 95%CI: 0.17–0.81) and good practice (aOR: 0.48, 95%CI: 0.21–0.89). Moreover, respondents with a family member ≤4 were more likely to have good practices regarding food safety (aOR: 1.85, 95%CI: 1.13–3.03) (Table 6).

Knowledge showed a significant moderate positive correlation with attitude (Spearman's rho (*ρ*): 0.446, *P* < 0.001) and practice (*ρ*: 0.578, *P* < 0.001). In addition, attitude showed a significant moderate positive correlation with practice (*ρ*: 0.557, *P* < 0.001) (Table 7).

## 4. Discussion

This study was conducted on the KAP of food safety among rural households and included 400 women aged ≥18 years who are usually involved in food preparation. We noted that participants had an average score in the knowledge and above average scores in the attitude and practice domains. However, young, educated women and those from a higher socioeconomic group had significantly higher scores in all domains compared to their counterparts.

We found that almost all respondents (99%) knew that their hands should be washed before handling food, which is similar to that found among street food vendors in the Jashore region, Bangladesh (100%) [14], and food handlers working in canteens in Malaysia (100%) [21], but higher than that among chotpoti vendors (54%) in Dhaka, Bangladesh [22], and students (98.2%) in Malaysia [23]. However, in the study conducted in canteens in Malaysia, the majority (84%) of the respondents had knowledge that raw and cooked food should not be cut on the same cutting board, which was higher than that found in the present study (64.3%) [21]. Similarly, maximum chotpoti vendors in Dhaka 17 (99.1%) had knowledge about keeping preparation places clean. However, we found this to be lower among the women in households in rural Bangladesh (88.8%). In a study conducted on food handlers in military hospitals in Jordan, about 93.5% of the respondents knew that raw food should be kept separate from cooked foods and 96% of the participants knew that fruits and vegetables must be washed before eating, which was similar to our study (94.8% and 99.3%) [4].

In the present study, 88.8% knew that wiping cloth can spread microorganisms, which was higher than in a previous study conducted on street-cooked food handlers (SCFHs) in North Dayi District, Ghana (67.3%). Similarly, approximately four-fifths of food handlers in both of these studies agreed that keeping the kitchen surface clean reduces the risk of illness [24]. However, students in Turkey (95.2%) had a higher agreement regarding kitchen cleanliness compared to our study participants [25]. In our study, 69.2% of respondents agreed that keeping raw and cooked food separate helps prevent illness, which appears to be very low compared to that found among food handlers in the schools of Camaçari, Brazil (98.8%) [26]. However, the hand washing practice before or during food preparation among our respondents (90.8%) was better than that found among home cooks of rural Bangladesh (64%) in a previous study [27] and among adult consumers in Turkey (82%) [28].

We found that 83.3% of the respondents knew that refrigerating food slows bacterial growth, which was higher than a previous study conducted on food handlers in Slovenia (63.4%) [29]. In addition, almost 83.5% knew that cooked food should be served hot, similar to a study conducted in residential units in Singapore (88.3%) [30]. Concordant to their findings, more than three-quarters of the respondents agreed that the use of different knives and cutting boards for raw and cooked food is beneficial for our health. Another concordant finding was the practice of checking and throwing beyond the expiry date in both studies (93.5% in the present study vs. 97.1% in the Singapore study).

In a study conducted among 400 women involved in food preparation in urban households of Karnataka, India [31], the mean knowledge score of food safety was 7.1 ± 1.5 (total score, 11), the mean attitude score was 15.45 ± 1.65 (total score, 18), and the mean practice score was 30.18 ± 4.21 (total score, 40), indicating that the knowledge score was average and attitude and practice scores were above the average of the possible scores similar to our study. This similarity could be explained by the fact that women in the Indian subcontinent share comparable characteristics in many aspects. Moreover, similar to the Indian study, we found that the average scores in all three domains were significantly higher among young women, which probably reflects the latest developments in food safety knowledge and higher educational attainment in the younger generation (as education increases awareness).

Compared to a previous study [20] conducted among the urban households of two megacities of Bangladesh, we found a lower proportion of good knowledge scores among our respondents (78.77% vs. 33.75%), indicating a lack of full awareness of the different safety measures recommended by WHO among rural women. Despite this, they had a good attitude and practice probably because of the shared experience of the previous generations handed down to their offspring.

Food safety and hygiene procedures are influenced by various factors. While some studies have found that knowledge of food handling is significantly connected to food handling practices [11, 32, 33], others in India and Bangladesh have found that food hygiene practices are associated with food handlers' educational status [34, 35]. We discovered in this study that educated participants had significantly more KAP scores than illiterate ones. In addition, we also found that knowledge was positively associated “with attitude and practice of food-safety among our respondents.”(I can edit the sentence. But I don't know why it gets back to previous state.”.

However, the most important independent determinant of good KAP scores was the economic status of the respondent. We noted that a lower economy with a marginal family income of <5,000 BDT per month was significantly associated with poor food-safety KAP, probably because a poor economic condition might not allow a rural woman to have the luxury of knowing and maintaining all the required food-safety measures, as they often do not get the opportunity to have quality education and have to live in crowded space. Interestingly, we also noted that living in a family with >4 members is independently associated with poor food handling practices.

It is difficult to make exact comparisons of the findings of different epidemiological studies on KAP of food safety measures conducted at various times due to the heterogeneity of the population (restaurants, mess of different campuses, street vendors, etc.), different sociocultural patterns in cooking practices across regions and nationality, and different tools used for outcome measures. However, a broad overview of the patterns shows that food handlers in our rural households, compared to others such as food vendors or restaurant food handlers [19], are generally aware of food-safety practices and are trying to implement their knowledge in day-to-day practice.

### 4.1. Current Measures to Increase Food Safety Practice

Food safety needs to be ensured at the national and international levels. Hence, individual countries at the national level have their strategies and policies for food safety. These are prepared in line with the guidelines [6, 12] laid out by WHO and Food and Agriculture Organization (FAO). The Government of the People's Republic of Bangladesh has incorporated an objective in the National Nutrition Policy 2015 to ensure the availability of adequate, diversified, and quality safe food and promote healthy feeding practices [36]. It emphasized access to adequate, diverse, and safe food and good eating habits. FAO implemented a food safety program entitled “Improving Food Safety in Bangladesh,” which is run in Bangladesh to help promote public health through an efficient and effective food safety control system [36]. Courtyard counseling meetings to improve hygienic food cooking practices were considered and were found to enhance knowledge about safe food [37].

### 4.2. Limitation

This study was one of the earliest attempts to evaluate KAP about food safety among food handlers in the rural households of developing Bangladesh using the WHO “five keys for food safety” tool. However, it had certain limitations. Food safety practices are more difficult to evaluate because of the chance of recall bias. The day-to-day practices on food safety could not be evaluated due to the cross-sectional nature of this study. The investigator did not directly observe the practices regarding food safety. Hence, this study is prone to bias because of the respondents' self-reported practices and behaviors.

### 4.3. Recommendations

Based on the findings of our study, we have the following recommendations for improving food safety knowledge and practices:

- Incorporating community clinic-based training and counseling programs on food safety practices in national nutrition and food safety plans
- Running audio-visual and social media-based awareness-raising programs about food safety practices
- Implementing monitoring and evaluation strategies to ensure compliance with key food safety areas
- Conducting community-based intervention research to discover innovative ways of increasing food-safety practice

## 5. Conclusion

The results from this study showed that KAP scores regarding food safety were relatively satisfactory among rural women handling food in households. Because knowledge of cooking thoroughly and keeping food at a safe temperature was found lower among the respondents, training and education on food safety measures could improve these areas. In addition, a low economic status was found to negatively impact KAP regarding food safety. Food handlers trained on the five keys to food safety by WHO can apply their knowledge during food preparation, thereby preventing foodborne illness prevalent in the low-resource countries. In addition, improving economic conditions could improve food safety measures. We recommend further interventional studies to determine the outcome of different interventions in improving food-handling practice in the household of this country.

## Figures and Tables

**Table 1:** Sociodemographic characteristics of the study participants.

Variables name	Category	
Age (years)		42.09 ± 12.96

Age group (years)	18–25	43 (10.8)
	26–35	112 (28.0)
	36–45	97 (24.3)
	46–55	87 (21.3)
	>55	61 (15.3)

Religion	Muslim	398 (99.5)
	Hindu	1 (0.3)
	Christian	1 (0.3)

Marital status	Married	321 (80.3)
	Unmarried	2 (0.5)
	Widow	77 (19.3)

Education	Illiterate	142 (35.5)
	Primary	178 (44.5)
	Secondary	79 (19.8)
	Undergraduate	1 (0.3)

Occupation	Housewife	218 (54.5)
	Farmer	75 (18.8)
	Worker	64 (16.0)
	Employee	23 (5.8)
	Others	20 (5.0)

Family type	Nuclear family	332 (83.0)
	Joint family	68 (17.0)

Family members (persons)	2	54 (13.5)
	3	85 (21.3)
	4	142 (35.5)
	>4	119 (29.8)

Type of house^*∗*^	Kacha	289 (72.3)
	Semipakka	98 (24.5)
	Pakka	13 (3.3)

Family toilet type^*∗*^	Kacha	16 (4.0)
	Pakka	384 (96.0)

Family income (BDT/Per Month)	Low (below 5,000 taka)	60 (15.0)
	Middle (5,000–10,000 taka)	153 (38.3)
	High (above 10,000 taka)	187 (48.8)

Data were expressed as mean ± SD and *n* (%) where appropriate. ^*∗*^Pakka means walls, including roofs made of concrete; semipakka means only walls made of concrete; and Kaccha means walls and roofs made of materials other than concrete (e.g., corrugated galvanized sheets).

**Table 2:** Distribution of the study participants according to the knowledge of the World Health Organization's five keys for food safety (*n* = 400).

Knowledge of WHO's five keys for food safety	*n* (%)
Key one: keep clean	
Wash hands before food handling (true)	396 (99)
Wiping cloth can spread microorganisms (true)	355 (88.8)

Key two: separate raw and cooked food	
The same cutting board could be used for raw and cooked food (false)	257 (64.3)
Raw food needs to be stored separately from cooked food (true)	379 (94.8)

Key three: cook thoroughly	
Cooked food need not be thoroughly reheated (false)	241 (60.3)

Key four: keep food at a safe temperature	
Cooked meat can be left at room temperature overnight (false)	50 (12.5)
Cooked food should be kept very hot before serving (true)	334 (83.5)
Refrigerating food slows bacterial growth (true)	333 (83.3)

Key five: use safe water and raw materials	
Safe water can be identified by the way it looks (false)	107 (26.8)
Wash fruit and vegetables (true)	397 (99.3)

WHO: World Health Organization.

**Table 3:** Distribution of participants according to the attitude toward the World Health Organization's five keys for food safety (*n* = 400).

Attitude toward WHO's five keys for food safety	Agree, *n* (%)	Not sure, *n* (%)	Disagree, *n* (%)
Key one: keep clean			
Frequent hand washing during food preparation	309 (77.3)	66 (16.5)	25 (6.3)
Keeping the kitchen surface clean reduces risk of illness	326 (81.5)	71 (17.8)	3 (0.8)

Key two: separate raw and cooked food			
Keeping raw and cooked food separate helps prevent illness	279 (69.8)	116 (29)	5 (1.2)
Using different knives and cutting boards for raw and cooked food	314 (78.5)	73 (18.3)	13 (3.3)

Key three: cook thoroughly			
Soup and stews boiled for safety	308 (77)	88 (22)	4 (1)

Key four: keep food at a safe temperature			
Thawing food in cool place is safer	220 (55)	169 (42.3)	11 (2.8)
Unsafe to leave cooked food out of refrigerator >2h	180 (45)	192 (48)	28 (7)

Key five use safe water and raw materials			
Inspection food for freshness and wholesomeness	384 (96)	15 (3.8)	1 (0.3)
Important to throw food beyond the expiry date	374 (93.5)	19 (4.8)	7 (1.7)

**Table 4:** Distribution of participants according to the practices of World Health Organization's five keys for food safety (*n* = 400).

Practices of WHO's five keys for food safety	Always (%)	Most of the time (%)	Occasionally (%)	Never (%)
Washing hands before or during food preparation	90.8	8.5	0.8	0
Cleaning the kitchen surface and equipment for food preparation before reusing for other food	83.3	15.3	0.5	1.0
Using separate utensils and cutting board when preparing raw and cooked food	66.5	24.5	5.8	3.3
Separating raw and cooked food during storage	84.3	11.8	3.5	0.5
Checking that meats are cooked thoroughly by ensuring that the juices are clear or by using a thermometer.	76.5	14	8.3	1.3
Reheating cooked food until it is piping hot throughout	40.3	40.3	16.5	3.0
Thawing frozen food in a refrigerator or other cool places	41	28	20.5	10.5
Storing leftovers in a cool place within two hours	39.5	25	21.3	14.2
Checking and throwing away food beyond its expiry date	95.5	0	0	4.5
Washing fruits and vegetables with safe water before eating them	92.5	6.3	1.3	0

**Table 5:** Knowledge, attitude, and practice scores by participant characteristics.

Variable	Knowledge score	*P -*value	Attitude score	*P*-value	Practice score	*P*-value
Age group (years)						
18–25	7 (7–8)	<0.001	16 (15–17)	<0.001	27 (24–29)	<0.001
26–35	7 (7–8)		16 (14–17)		26 (24–27)	
36–45	7 (7–8)		16 (15–17)		26 (24–28)	
46–55	7 (6–8)		16 (14.5–17)		25 (22–27.5)	
>55	6 (4–7)^abcd^		12.5 (7–16)^abcd^		21.5 (11–26)^abcd^	

Marital status						
Single	7 (5–7)	<0.001	15 (8–17)	0.002	23 (14–27)	<0.001
Married	7 (7–8)		16 (15–17)		26 (23–28)	

Education						
Illiterate	7 (5.5–8)	<0.001	16 (8–17)	0.012	24 (13–27)	<0.001
Literate	7 (7–8)		16 (15–17)		26 (24–28)	

Occupation						
Unemployed/inactive	6.5 (5–7)	0.016	11 (7–16)	0.005	18.5 (11–25.5)	<0.001
Employed/active	7 (6–8)		16 (14–17)		26 (23–28)	

Family type						
Nuclear	7 (6–8)	0.604	16 (14–17)	0.580	26 (22–28)	0.172
Joint	7 (6–8)		15 (13.5–17)		25 (19–27)	

Family members						
≤4	7 (6–8)	0.188	16 (14–17)	0.371	26 (23–28)	0.025
>4	7 (6–8)		16 (10–17)		25 (19–28)	

House type^*∗*^						
Kacha	7 (6–8)	<0.001	16 (14–17)	0.005	25 (22–28)	0.134
Pakka/semipakka	7 (7–8)		16 (15–17)		26 (24–28)	

Monthly family income (BDT)						
<5,000	6 (4–7)	<0.001	11 (7–16)	<0.001	21 (11–26)	<0.001
5,000–10,000	7 (7–8)^a^		16 (15–17)^a^		26 (24–28)^a^	
>10,000	7 (7–8)^a^		16 (14–17)^a^		26 (24–28)^a^	

^*∗*^Pakka means walls, including roofs made of concrete; semipakka means only walls made of concrete; and Kaccha means walls and roofs made of materials other than concrete (e.g., corrugated galvanized sheets).

**Table 6:** Univariate and multivariable logistic regression analysis exploring factors associated with good knowledge, attitude, and practice among participants.

Variable	Knowledge			Attitude		Practice	
	Reference category	Crude OR (95%CI)	Adjusted OR (95%CI)	Crude OR (95%CI)	Adjusted OR (95%CI)	Crude OR (95%CI)	Adjusted OR (95%CI)
Age in years (>55)	≤55	0.41 (0.21–0.82)	0.69 (0.31–1.54)	0.16 (0.09–0.28)	0.38 (0.17–0.81)	0.21 (0.12–0.38)	0.48 (0.21–0.89)
Education (literate)	Illiterate	1.38 (0.89–2.15)	0.84 (0.50–1.42)	3.39 (2.04–5.64)	1.31 (0.68–2.55)	2.25 (1.46–3.49)	1.02 (0.59–1.78)
Occupation (employed/active)	Unemployed/inactive	2.10 (0.70–6.42)	1.17 (0.35–3.94)	4.51 (1.81–11.23)	1.59 (0.51–4.89)	4.50 (1.75–11.58)	2.04 (0.69–6.00)
Marital status (married)	Single	1.79 (1.02–3.14)	1.07 (0.55–2.10)	3.14 (1.82–5.44)	0.78 (0.37–1.68)	2.89 (1.74–4.80)	1.18 (0.62–2.26)
Number of family members (≤4)	>4	1.17 (0.73–1.86)	1.03 (0.64–1.68)	2.09 (1.25–3.49)	1.70 (0.94–3.08)	2.19 (1.39–3.45)	1.85 (1.13–3.03)
House type^*∗*^ (pakka/semipakka)	Kacha	1.76 (1.26–2.78)	1.48 (0.93–2.37)	2.23 (1.17–4.23)	1.27 (0.63–2.58)	1.92 (1.15–3.21)	1.36 (0.79–2.36)
Monthly family income in BDT (≥5,000)	<5,000	4.27 (2.05–8.90)	3.51 (1.55–7.98)	9.59 (5.40–17.07)	5.82 (2.89–11.70)	5.48 (3.18–9.45)	3.18 (1.67–6.07)

^*∗*^Pakka means walls, including roofs made of concrete; semipakka means only walls made of concrete; and Kaccha means walls and roofs made of materials other than concrete (e.g., corrugated galvanized sheets). OR: odds ratio. ORs significant at *P* < 0.05 level are shown in bold.

**Table 7:** Spearman correlations among knowledge, attitude, and practice scores.

	Knowledge	Attitude	Practice
Knowledge	1.000	0.446^*∗*^	0.578^*∗*^
Attitude	0.446	1.000	0.557^*∗*^
Practice	0.578^*∗*^	0.557^*∗*^	1.000

Data represent Spearman's rho. ^*∗*^Significant at *P* < 0.001.

## Data Availability

Data are available upon reasonable request.
